# Supplement to the Doctrine of the Circulation. Second Part. Ciliary Motions, Life of Blood-Globules, Doctrine of Monads

**Published:** 1837-04

**Authors:** 


ART. II.—*Supplemente zur Lehre vom Kreislaufe. II. Heft. Flimmerbewegungen, Leben der Blutsphären, Monadenlehre.* Von Dr. A. F. I. C. MAYER, Ord. öff. Professor der Anatomie und Physiologie zu Bonn, &c.—*Bonn*, 1836. 4to. pp. 55.

*Supplement to the Doctrine of the Circulation. Second Part. Ciliary Motions, Life of Blood-globules, Doctrine of Monads.* By Dr. A. F. I. C. MAYER, Professor of Anatomy and Physiology at Bonn.—*Bonn*, 1836. 4to. pp. 55.

THE past reputation of Professor Mayer, and the services which he has, in various ways, rendered to medical science, naturally claim attention for any work that bears his name. It was therefore with sincere regret that we rose from the perusal of the pamphlet now before us, as we feel that, in discharging our duty to our readers in making them acquainted with its contents, it would be impossible not to pass a severe sentence on its author. In truth, with the exception of one single observation, which, however, is an important one, the whole memoir is nothing less than a tissue of extravagancies; and it is painful to observe that the errors of the learned writer are not casual, but are the cherished and systematic errors of a sect or school.

Dr. Mayer belongs to a class of physiologists, a pretty large one in Germany, whose zeal, and (not unfrequently) whose accuracy as observers, enable them from time to time to add materially to the stock of real knowledge, but who, as reasoners, or rather speculators, too often follow headlong wherever fancy leads them, throwing aside common sense and all such gross encumbrances as might stay them within the vulgar sphere of rational philosophy. The works of such men present a curious combination. On the one hand, we see evidence of actual observation, often to a great extent, displaying great labour, and often much ingenuity; on the other, we see these observations wrongly interpreted, the most obvious circumstances overlooked, and facts in general exaggerated, pared down, or distorted in favour of some wild and extravagant speculation, to which every thing is sacrificed. The doctrine of Monads, "Monadenlehre," is Dr. Mayer's idol; and assuredly he worships and serves it like a faithful high priest. An uninitiated, or rather unsophisticated English reader, on perusing Dr. M.'s production, would conclude either that it was intended as an amusing piece of ridicule, or that, if serious, the author was nothing but a wild enthusiast. But Dr. M. is serious, and nevertheless he is something better than a mere enthusiast; for it would be unjust to deny that on this, as well as on other occasions, he has contributed original and not altogether unimportant observations. To speak more particularly, however, of the contents of the paper:

Dr. M. has discovered the vibratory, or, as we would call it, ciliary motion on the pericardium, pleura, and peritoneum of the frog. This observation we find to be perfectly correct. He states, moreover, that the phenomenon is confined to small patches on the surface, placed at some distance from each other; but on this point we are disposed to differ from him; for, although there are some parts of the peritoneum on which we could not perceive it, and on which it may perhaps not exist, yet there are large regions on which the motion is seen over the entire space without interruption; and we have not been able to see the insular arrangement which he describes. The phenomenon had not been previously seen on serous membranes, unless we consider as such the so named peritoneum, or membrane lining the visceral cavity of echinodermata and annelida, on which it had been observed by Professor Sharpey.

Dr. Mayer next endeavours to prove that the motion is not produced by cilia. He denies the existence of any such organs in the majority of instances, and particularly on the serous surfaces, ascribing the appearances taken by others for cilia to optical illusion, and attempting to establish his own opinion by means of several very palpable *non sequiturs.* Assuming that he has, in these cases, proved the absence of cilia, he next deals with those instances in which he cannot deny their existence. Here he endeavours to show that the cilia are not the agents, but are moved accidentally and passively. In this attempt he confounds the cilia with villi, and tells us gravely that, as villi may be seen on membranes where the motion in question never shows itself, they are not essential when they happen to coexist with it. From the gross mistake of confounding two things so different, we may know what weight to concede to the arguments founded on it; from this, also, any one who is familiar with the appearance of the cilia, as seen in several different animals, will be able to appreciate the extent and accuracy of Dr. M.'s observations: indeed, we are satisfied from this that he has not studied the phenomena extensively among the invertebrate tribes. But, not venturing to discard the very obvious agency of cilia in the Vorticellæ and Infusoria, he declares that the phenomena occasioned by the cilia in these animals are quite of a different character from that of which he is treating. On this last point we may remark, that undoubtedly, when the cilia are very minute, they can with difficulty be perceived as separate filaments, especially while in rapid motion; but, even admitting that this difficulty amounted in some cases to an impossibility, yet the character of the motion which these organs collectively exhibit is precisely the same as in those instances in which they are large and easily recognized individually; and it is easy to trace the intermediate gradations which connect the two extremes, and show that in both the phenomena are essentially of the same kind. As regards serous membranes, on which Dr. Mayer positively and confidently denies the existence of cilia, we must say that we find no difficulty in detecting these organs, though they are very minute, on the serous membranes of the frog; and we know that Dr. Sharpey had previously seen them on the corresponding membranes of some of the Invertebrata. As to the share of the cilia in the motion, we refer to the arguments advanced by the gentleman just named, in an excellent article in the Cyclopædia of Anatomy, (Article *Cilia*, Part II. sect. 10 and 11.)

Having, as he supposes, got rid of the cilia, Dr. Mayer next reveals to us his own views as to the nature of the motion. He begins by announcing a most important discovery of his own, which he wonders never occurred to anybody else; viz. that when the mucous secretion is scraped off from the membranes in question, the masses of mucus exhibit the vibratory motion. Now, every one engaged in such enquiries has seen the appearance to which he alludes, but has been contented to explain it by supposing that some fragments of the membrane, bearing a few cilia, had been detached by the scraping, and, continuing their motion, had imparted the same to the masses of mucus in which they were entangled. Not so Dr. Mayer. From this fact and some other equally weighty considerations, he concludes that the vibratory motion is produced by a peculiar substance, named by him "vibratory matter," (*zitterstoff*,) which adheres to the surfaces on which the phenomenon shows itself. He conceives this substance to pervade the whole animal body, to be "the source of vital formation,"—"the fountain from which life flows,"—"itself the product of life, but yet the primitive or original formative substance, and therefore again productive." While it forms part of the tissues of the body, its energies are fettered, its independent living powers controlled; but on the surface of the organs it exercises its own innate powers, manifesting them by its vibrations, and still more so when it is separated altogether from the tissues; for then it forms new and independent living existences. This mysterious substance consists of innumerable little globular *monads*, dwelling in a clear slimy matter, forming, according to the author's comparison, a kind of galaxy. The monads are not idle; they continually whirl round their somewhat elongated axis, like spinning tops, and give rise to the apparent vibration. Dr. Mayer finds that they grow bigger while thus employed, and seriously tells us that, when first observed, they are about the ten-thousandth of a line in size, but afterwards grow to a five-thousandth; he does not say when this growth stops or when it begins. The monads, however, do not always keep spinning thus; they detach themselves, and then become independent infusoria, able to shift for themselves in the world. The blood-globules are infusoria, originally monads, we presume, but now elevated to the dignity of "Biospheres." These biospheres have a great deal to do with the vibratory matter; in short, they feed on it, devouring the unlucky fry of monads; and Dr. Mayer's description of a biosphere taking a repast is very entertaining. He tells us that, on folding a piece of membrane, he has seen neighbouring blood-globules approach the vibrating edge, and leave it again; and thus far, indeed, things have been seen by many others: but Dr. M. perceives that the biosphere plunges among the crowd of monads, like a wolf among a flock of sheep, and swallows a few; and he finds that a biosphere, after thus regaling itself, becomes larger, and the monads may be seen in its inside! Like his predecessors, who support the theory of larger animals being an aggregation of monads, Dr. Mayer is also of opinion that such larger animals are really formed at first by the union of a parcel of monads; but he enjoys the great advantage of having often witnessed the process of formation going on under his own eyes. What simple men like ourselves regard as detached shreds of membrane, or of membrane and mucus, moved about by cilia, Dr. M. finds out to be new animals in the act of being formed by the aggregation of a pinch of monads, or, what is the same thing, by a portion of "zitterstoff;" but, as he seems doubtful whether such a new creature is likely to subside into the form of any existing species, he names it "Unthier," or "Monster." After telling wonderful tales about this Promethean production, which he can make at will, and about the biospheres and their ogre-like propensities, he concludes his nursery-tale by informing us, with the greatest self-complacency, that the secret of animal formation is now revealed; and, of course, that the *Monadenlehre*, combined with his own discovery of the *Zitterstoff*, will serve as a sort of talisman, before which every hidden thing in the living economy will be laid open.

Such is a specimen of Professor Mayer's physiological doctrines promulgated in the present work, which, in justice to the rational physiology of Germany, of which no one can think higher than ourselves, we have felt it our duty to expose. Although such doctrines are justly appreciated by some of the author's more philosophic countrymen, yet similar—and even, if possible, greater—absurdities are by no means peculiar to him, but are shared by many individuals distinguished by their past labours and by their talents. We are the more anxious to characterize them as we have done, because we think there is a tendency, among certain enquirers at present, in our own country, to countenance visionary speculations in science, without regard to those laws of legitimate induction, for which our best English writers have now for so many years been happily distinguished. There are some men who cannot cross the street without meeting with an adventure or seeing a wonder; others, again, contrive to pass through life without encountering anything very marvellous. Dr. Mayer is evidently one of the former class. The phenomena presented by the cilia are curious enough in themselves, as seen by plain observers; and there was no necessity to invent a fairy-tale in order to excite our interest.

For a complete account of all that is yet known on the subject of ciliary motions, we refer our readers to the elaborate article, in the first volume of the "Cyclopædia of Anatomy and Physiology," already referred to. The only addition of importance to the information contained in that paper that has come to our notice, is the discovery, by

Purkinje and Valentin, of the motions on the lining membrane of the ventricles of the brain, which, together with some observations of Siebold, are noticed in another part of the present Number. (See Foreign Selections.)

